# Nationwide analysis of COVID-19 complications, outcomes, and costs of childbirth in Spain

**DOI:** 10.3389/fmed.2025.1548245

**Published:** 2025-05-08

**Authors:** Blanca Álvarez-del Río, Laura Sánchez-de Prada, Irene Arroyo-Hernantes, F. Javier Álvarez, Eduardo Tamayo, Eduardo Gutiérrez-Abejón

**Affiliations:** ^1^Facultad de Medicina, Universidad de Valladolid, Valladolid, Spain; ^2^BioCritic, Grupo de investigación de Biomedicina en Cuidados Críticos, Valladolid, Spain; ^3^Hospital Clínico Universitario de Valladolid, Valladolid, Spain; ^4^Centro de Investigación Biomédica en Red de Enfermedades Infecciosas (CIBERINFEC), Instituto de Salud Carlos III, Madrid, Spain; ^5^Dirección Técnica de Farmacia, Gerencia Regional de Salud de Castilla y León, Valladolid, Spain; ^6^Facultad de Empresa y Comunicación, Universidad Internacional de la Rioja (UNIR), Logroño, Spain

**Keywords:** COVID-19, pregnancy, childbirth, delivery, hospital direct costs, costs per patient, Spain, costs

## Abstract

**Introduction:**

Pregnant women are considered a vulnerable group for COVID-19 with an increased risk for complications. The objective of this study is to describe in-hospital mortality, pregnancy outcomes, and direct hospital costs associated with COVID-19 in women at the time of childbirth.

**Methods:**

This retrospective nationwide population-based registry study collects data on complications, outcomes, and direct hospital costs from women hospitalized for childbirth, recorded in the Minimum Basic Data Set obtained from the National Surveillance System for Hospital Data in Spain between 2020–2022. Hospitalization characteristics, complications related to pregnancy and childbirth, outcomes, and hospitalization costs are compared between COVID-19-positive and non-COVID-19 women at the time of childbirth.

**Results:**

A total of 779,387 women were admitted between 2020 and 2022 with a record of childbirth in Spanish hospitals. Of these, 15,792 (2.06%) had COVID-19 at the time of delivery. These women had a longer length of stay (3.53 days), higher rates of intensive care unit (ICU) admission (2.53%), ventilation/intubation (0.91%), and in-hospital mortality (0.06%) (*p* < 0.0001). This group also exhibited higher rates of spontaneous premature onset of labor (7%) and postpartum hemorrhage (3.45%), as well as a higher rate of labor induction (6.27%) (*p* < 0.001). Additionally, a higher single stillbirth rate (0.73%) was found among COVID-19-positive women (*p* = 0.0002). A significant higher risk of postpartum hemorrhage (OR = 1.14), embolism (OR = 7.98), acute respiratory distress syndrome (OR = 35.5), temporary tracheostomy (OR = 4.89), ventilation/intubation (OR = 6.85), and single stillbirth (OR = 1.32) was found in COVID-19 women (*p* < 0.05). The mean cost per patient was €4,066.48, 25.06% higher than that for non-COVID-19 women (*p* < 0.0001). Stratification by age showed an increasing trend in costs with age, reaching €6,492.12 in women ≥45 years old, where the ICU admission rate reached 8.09%.

**Conclusion:**

These findings show that COVID-19 at the time of childbirth occurs in 2 out of every 100 cases and increases the risk of complications related to pregnancy and childbirth, as well as mortality and hospitalization costs. These data are related to SARS-CoV-2 variants circulating from 2020–2022, and current variants could give different risks. Our evaluation should be useful for health authorities to allocate resources and professionals to implement preventive measures, such as vaccination and screening, due to the increased morbidity, mortality and costs in this group.

## 1 Introduction

COVID-19, caused by the novel severe acute respiratory syndrome coronavirus (SARS-CoV-2) emerged in 2019, resulting in a pandemic by March 2020 (1). The constant mutation of the virus and the emergence of new variants have challenged the countries worldwide in their attempt to control the pandemic (2, 3). By the end of 2022, nearly 730 million cases and 6.7 million deaths had been caused by the virus (4).

On one hand, pregnant women belong to high-risk group regarding COVID-19. Many studies have focused on deciphering the impact of COVID-19 infection on both mother and fetus at different stages (5, 6) which still remains uncertain. On the other hand, the pandemic has resulted in an unprecedent global health and macroeconomic crisis affecting countries worldwide (7, 8). Many studies have tried to assess the costs of COVID-19 hospitalizations and primary care (9), but none of them has focused on pregnancy and childbirth. Using nationwide records, the aim of this study is to evaluate the impact of COVID-19 infection on maternal morbidity, mortality, pregnancy outcomes, and its economic repercussion in Spain during 2020–2022.

## 2 Methods

### 2.1 Study design

We conducted a nationwide hospital registry-based retrospective cohort study on pregnant women with a record of delivery in Spanish hospitals (public and private) during three consecutive years, starting from the beginning of the SARS-CoV-2 pandemic (2020–2022).

Inclusion criteria involved all pregnancies with a record of childbirth during 2020–2022. Exclusion criteria involved not having a record of childbirth. Underlying conditions were not an exclusion criterion.

Data were obtained from the Minimum Basic Data Set (MBDS) (10) collected by the National Surveillance System for Hospital Data in Spain and published by the Ministry of Health with a two-year lag. The MBDS is a clinical and administrative database completed at the time of discharge, with an estimated coverage of 99.5% of public and private Spanish hospitals. It provides encrypted information following the International Classification of Diseases 10th Revision, Clinical Modification (ICD-10-CM) (11). The study was approved by the Ethics Review Board (CEIm Area de Salud Valladolid Este, reference study PI-22–2855), and informed consent was waived due to the anonymous nature of data contained in the MBDS. We followed the CHEERS reporting guideline.

### 2.2 Measures

Pregnant women with a record of childbirth during 2020–2022 were selected. Patients were then divided into two groups according to confirmed COVID-19 diagnosis following ICD-10-CM (11) codes B97.29 (“Other coronavirus as the cause of diseases classified elsewhere “), and U07.1 (“COVID-19, virus identified”)upon admission.

Variables collected included age, sex, length of stay (LoS), in-hospital mortality, admission to the intensive care unit (ICU), ICU, LoS, ICU mortality, mechanical ventilation and ventilatory assistance. Comorbidities, complications, and outcomes were identified (Table 1). Supplementary Table 1 provide the ICD-10-CM codes used to identify comorbidities and complications in the included patients.

**Table 1:** Characteristics of hospitalized women at childbirth based on COVID-19 infection during 2020–2022.

	COVID-19 at childbirth	Non-COVID-19 at childbirth	*p*-value
No	15,742	763,645	
Mean Age (years)	31.58 (31.48–31.67)	32.27 (32.26–32.28)	<0.0001
Length of stay (days)	3.53 (3.25–3.80)	2.94 (2.93–2.95)	<0.0001
In-hospital mortality	9 (0.06)	31 (0.00004%)	<0.0001
ICU			
ICU admission	398 (2.53%)	4,670 (0.61%)	<0.0001
ICU mortality	2 (0.01%)	8 (0.00001%)	0.0035
ICU length of stay	6.89 (5.87–7.91)	1.24 (1.13–1.36)	<0.0001
Comorbidities			
Asthma	408 (2.59%)	20,461 (2.68%)	0.5164
Diabetes	37 (0.24%)	1,974 (0.26%)	0.6207
Chronic hypertension	18 (0.11%)	988 (0.13%)	0.6833
Chronic heart disease	126 (0.8%)	4,008 (0.52%)	<0.0001
Metabolic disorder	1,427 (9.06%)	71,817 (9.4%)	0.1523
Immunosuppression	58 (0.37%)	2,640 (0.35%)	0.6803
Blood disorder	306 (1.94%)	12,787 (1.67%)	0.0101
Neurologic disease	135 (0.86%)	4,888 (0.64%)	0.0009
Renal disease	33 (0.21%)	1,010 (0.13%)	0.0118
Pregnancy complications			
Preeclampsia and eclampsia	168 (1.07%)	7,596 (0.99%)	0.3864
Gestational diabetes	1,235 (7.85%)	61,547 (8.06%)	0.3353
Premature rupture of membranes	3,894 (24.74%)	196,318 (25.71%)	0.0059
Antepartum hemorrhage	34 (0.22%)	1,591 (0.21%)	0.9047
Abortion	4 (0.03%)	179 (0.02%)	0.9999
Spontaneous premature onset of labor	1,102 (7%)	41,114 (5.38%)	<0.0001
Induction of labor	987 (6.27%)	36,015 (4.72%)	<0.0001
Cesarean section	25 (0.16%)	1,882 (0.25%)	0.0339
Postpartum hemorrhage	543 (3.45%)	22,544 (2.95%)	0.0003
Other complications			
Acute renal failure	30 (0.19%)	339 (0.04%)	<0.0001
Acute respiratory distress syndrome	233 (1.48%)	169 (0.02%)	<0.0001
Embolism	29 (0.18%)	70 (0.01%)	<0.0001
Temporal tracheostomy	27 (0.17%)	10 (0%)	<0.0001
Ventilation/intubation	144 (0.91%)	178 (0.02%)	<0.0001
Shock	23 (0.15%)	330 (0.04%)	<0.0001
Outcomes			
Single livebirth	15,398 (97.81%)	746,843 (97.8%)	0.9207
Single stillbirth	115 (0.73%)	3,929 (0.51%)	0.0002
Twins, both liveborn	223 (1.42%)	12,310 (1.61%)	0.0578
Twins, one liveborn and one stillborn	2 (0.01%)	287 (0.04%)	0.1628
Twins, both stillborn	2 (0.01%)	137 (0.02%)	0.8529

No, number; ICU, intensive care unit. Values are expressed as absolute number (percentage) and mean (CI95%).

Hospital costs were calculated using all patient refined diagnosis-related groups (APR-DRG) data extracted from the MBDS. The DRG weights and costs are derived from a representative sample of hospitals that perform analytic accounting in our country and are periodically collected in the Spanish Record of Hospital Costs. Costs calculated through the APR-DRG are adjusted for severity and mortality risk based on patient characteristics, secondary diagnosis and procedures performed during the stay. It also takes into consideration type of hospital and number of beds to estimate costs individually. Additionally, the Spanish Record of Hospital Costs has data of individual costs at discharge since 2008 which allows to have updated data on weight and costs based on actual costs and real healthcare practice in the Spanish system (12). These costs were disaggregated by year, based on age groups (<20, 20–24, 25–29,30–34, 35–39, 40–45, >45 years old), ICU-admission, and autonomous regions. All costs were expressed in euros (€). The annual inflation was calculated used the INE tool and represented a 6.5% in 2021 and 12.1% in 2022 compared to 2020 (13). The annual currency equivalence between 2020–2022 was 1€ = 1.1261$ United States dollars (USD) (14).

### 2.3 Statistical analysis

Patients not fulfilling inclusion criteria were excluded. Results were reported as mean with 95% confidence interval (CI95%) for continuous variables. and as frequencies and percentages for categorical variables. According to data distribution, non-parametric tests were used. Differences between groups were assessed using Student T-test, Mann–Whitney U-test and Kruskal-Wallis adjusted with Bonferroni correction for multiple comparisons (*α* = 0.05) for continuous variables when appropriate. In order to adjust for cofounders, first an univariate regression was performed for each variable, using the presence or absence of COVID-19 as the response variable. Then, significant variables from the univariate analysis were then included in a multivariate regression. In the multivariate analysis, a stepwise procedure was applied to retain only the significant variables. Odds Ratio (OR) with the 95% Confidence Interval (CI95%) were calculated. Significance was considered at *p* < 0.05. All statistical analysis was conducted with R software version 4.3.2.

## 3 Results

### 3.1 Sample characteristics

**Figure 1:**
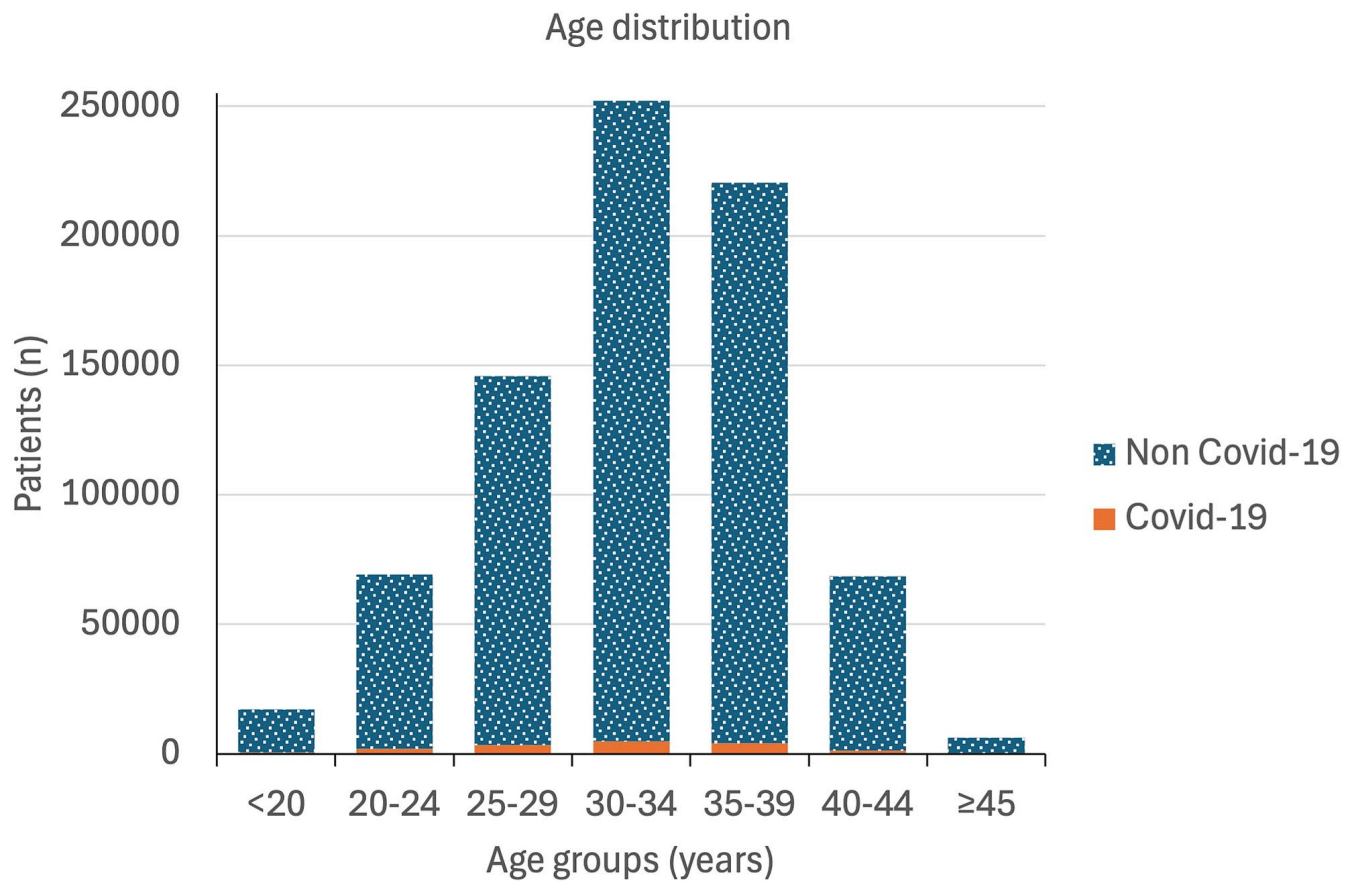
Age distribution of pregnant women at childbirth in Spain during 2020–2022.

A total of 779,387 women were admitted between 2020 and 2022 with a record of delivery in Spanish hospitals; of these, 15,792 (2.06%) had COVID-19 at the time of delivery. The prevalence was 1.24, 1.06 and 3.73% in 2020, 2021, and 2022, respectively. The mean age was 31.6 years old in COVID-19 patients and 32.3 years in non-COVID-19 patients (Table 1). Age distribution of patients has been represented in Figure 1.

### 3.2 Clinical characteristics and outcomes

Admission characteristics of both groups are described in Table 1. Pregnant women with COVID-19 at the time of delivery had significantly higher in-hospital mortality (0.06%) and LoS (3.53 days). A total of 2.53% of the women presenting with COVID-19 had to be admitted to the ICU and ICU mortality reached 0.01%. The mean ICU LoS was 6.89 days, and the ventilation/intubation rate was 0.91%. All of these variables significantly higher when compared to non-COVID-19 pregnant women (*p* < 0.0001) (Table 1).

Significant differences were observed in comorbidities, with higher rates of heart disease (0.8%), blood disorders (1.94%), neurological disorders (0.86%), and renal disease (0.21%) in the COVID-19 group (*p* < 0.05). Regarding complications, patients with COVID-19 exhibited significantly higher rates of acute renal failure (0.19%), acute respiratory distress syndrome (1.48%), embolism (0.18%), temporary tracheostomy (0.17%), ventilation/intubation (0.91%), and shock (0.15%) (*p* < 0.0001). Additionally, higher rates of pregnancy complications were observed in the COVID-19 group, including spontaneous preterm labor (7%), postpartum hemorrhage (3.45%), and labor induction (6.27%) (*p* < 0.001). Furthermore, a higher rate of single stillbirth (0.73%) was observed in COVID-19 patients (*p* = 0.0002) (Table 1).

**Figure 2:**
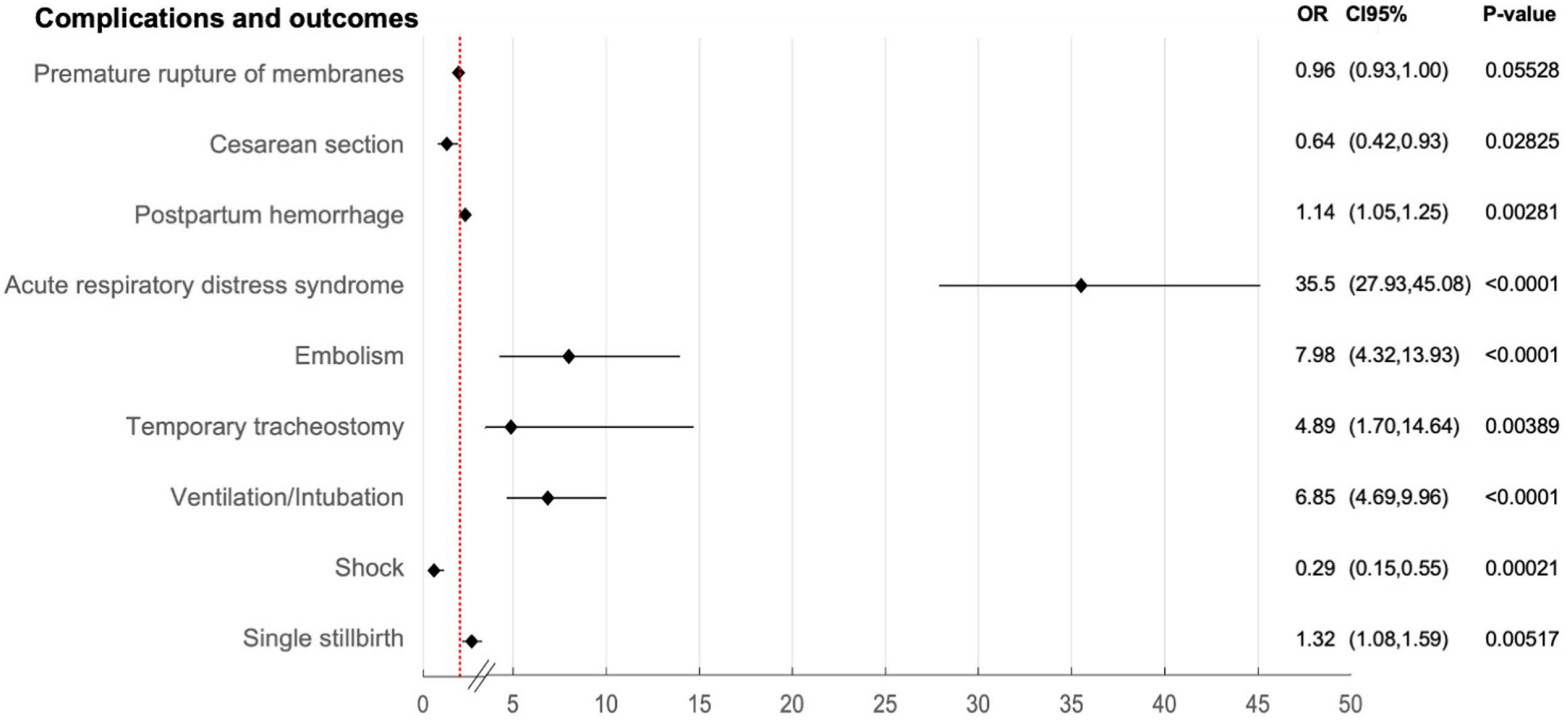
Association between COVID-19 in pregnant women at childbirth and complications and outcomes in Spain during 2020–2022.

The results of the multivariate model showed no significant association of comorbidities with the risk of COVID-19 (Supplementary Table 2). It can be observed that COVID-19 pregnant women were more likely to experience complications including acute respiratory distress syndrome (OR = 35.5), an embolism (OR = 7.98), a temporal tracheostomy (OR = 4.89) or the need of ventilation/intubation (OR = 6.85) (*p* < 0.05). However, the risk of cesarean section (OR = 0.64) or shock (OR = 0.29) was lower in this group (*p* < 0.05). The risk of postpartum hemorrhage (OR = 1.14) and single stillbirth were also significantly increased in the COVID-19 women (OR = 1.32) (*p* = 0.0051) (Figure 2).

### 3.3 COVID-19 hospitalization related costs in pregnant women at childbirth

The total cost of admissions for pregnant women with COVID-19 disease at the time of delivery was €64 million (€64,014,477), with a mean cost per patient of €4,066.48. This was significantly higher that the €3,251.59 per patient spent on non-COVID-19 patients (*p* < 0.0001) (Table 2). Admission to the ICU represented a higher mean cost per patient (€13,629.35) compared to non-COVID-19 women (€4,563.58) (*p* < 0.0001) (Table 2).

**Table 2:** Mean cost per patient (€) of hospitalization at childbirth in Spanish population based on COVID-19 infection during 2020–2022.

	COVID-19 at childbirth	Non-COVID-19 at childbirth	*p*-value
Year			
2020	4,204.26 (4,100.72–4,307.81)	3,316.12 (3,313.99–3,318.25)	<0.0001
2021	5,149.72 (4,944.97–5,354.47)	3,314.53 (3,312.31–3,316.76)	<0.0001
Adj-2021	5,484.45 (5,266.39–5,702.51)	3,529.97 (3,527.61–3,532.35)	
2022	3,621.95 (3,586.55–3,657.34)	3,116.56 (3,114.38–3,118.75)	<0.0001
Adj-2022	4,060.21 (4,020.52–4,099.88)	3,493.66 (3,491.22–3,496.12)	
Type of admission			
General admission	3,820.32 (3,796.85–3,843.79)	3,244.08 (3,242.95–3,245.21)	<0.0001
ICU admission	13,629.35 (11,962.94–15,295.77)	4,563.58 (4,472.87–4,654.29)	<0.0001
Age group			
<20	3,909.51 (3,743.54–4,075.48)	3,168.63 (3,161.98–3,175.29)	<0.0001
20–24	3,888.54 (3,781.30–3,995.79)	3,189.18 (3,185.42–3,192.93)	<0.0001
25–29	3,996.20 (3,889.81–4,102.6)	3,214.69 (3,212.09–3,217.28)	<0.0001
30–34	4,034.45 (3,931.43–4,137.48)	3,237.65 (3,235.47–3,239.83)	<0.0001
35–39	4,127.94 (4,025.19–4,230.69)	3,273.55 (3,271.09–3,276.02)	<0.0001
40–44	4,223.34 (4,040.09–4,406.59)	3,364.61 (3,359.11–3,370.12)	<0.0001
≥45	6,492.12 (4,449.70–8,534.54)	3,570.89 (3,550.83–3,590.89)	<0.0001
Total mean cost	4,066.48 (4,013.02–4,119.93)	3,251.59 (3,250.28–3,252.83)	<0.0001
Total cost	64,014,477	2,483,036,331	<0.0001

Adj-2021 and Adj-2022 represent the values adjusted for inflation to be comparable to 2020. ICU, intensive care unit. Values represent the mean cost in euros (CI 95%).

**Figure 3:**
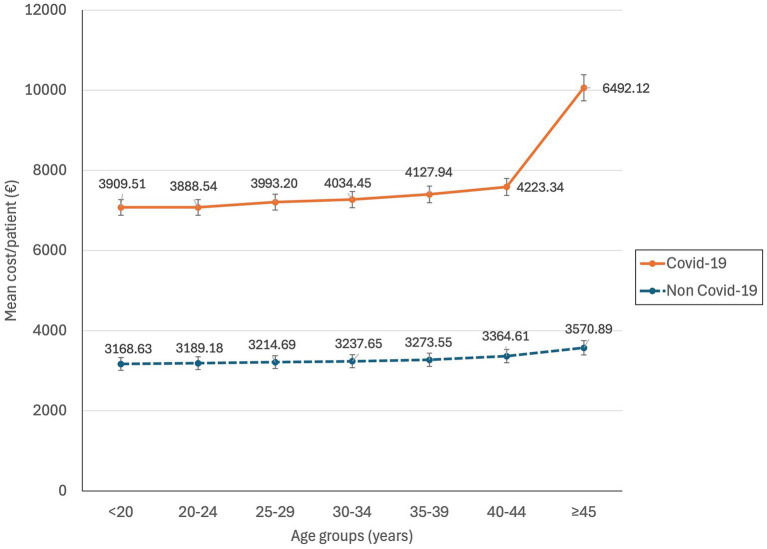
Mean cost per patient of hospitalization at childbirth according to age and COVID-19 infection in Spain during 2020–2022.

**Figure 4:**
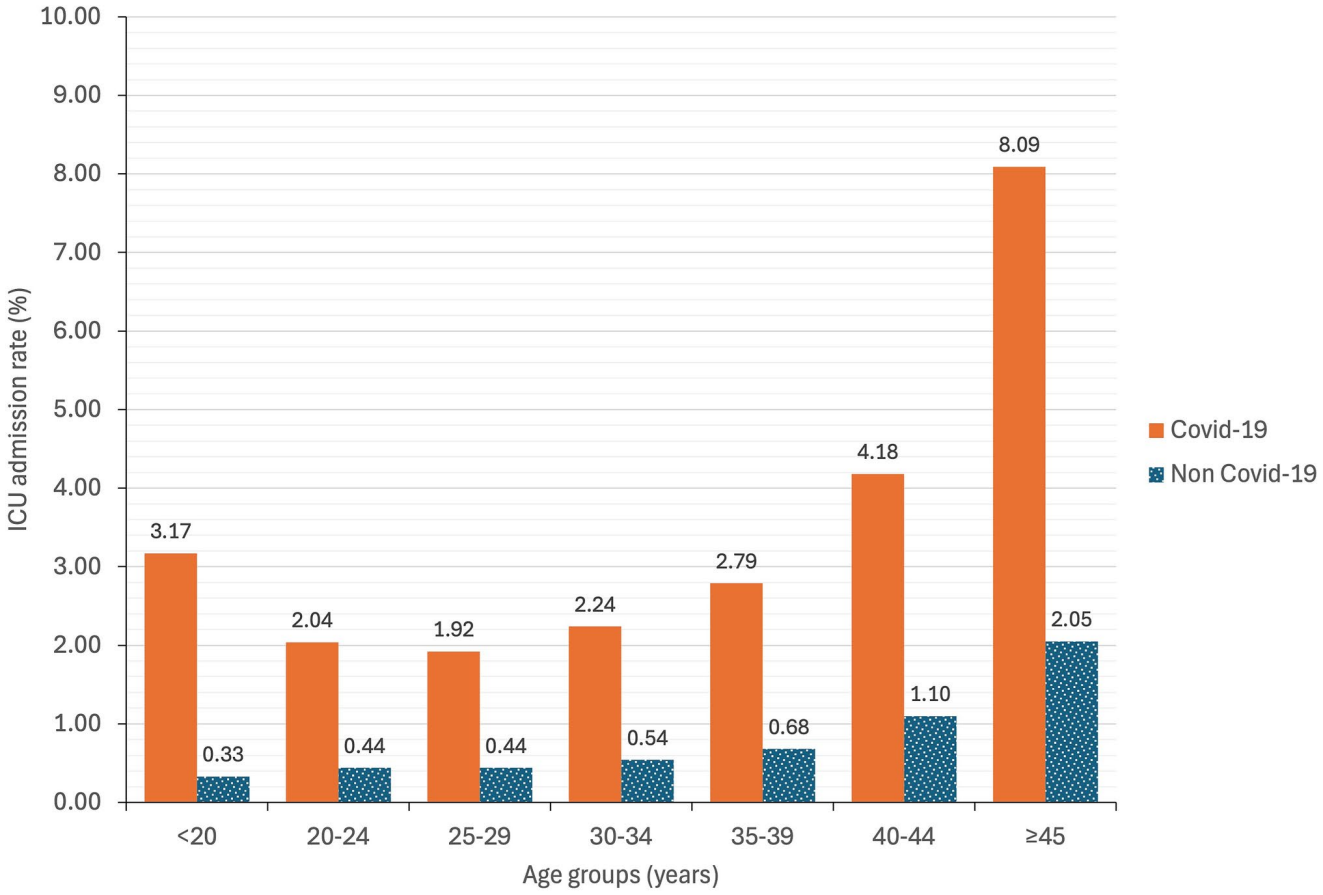
ICU admission rate of hospitalization at childbirth based on age groups and COVID-19 infection in Spain during 2020–2022.

Age distribution showed an increasing trend in expenditure per patient with age, which was significantly higher in COVID-19 patients compared to those without COVID-19 in the same age range (*p* < 0.0001) (Figure 3). ICU admission, shown in Figure 4, presented an increasing trend with age in non-COVID-19 patients. However, for COVID-19 patients, ICU admission showed an increasing trend starting from the 25–29 age range, but higher rates were found in patients under 20 years old.

Additionally, we performed a regional analysis of the mean cost per patient and ICU admission (Supplementary Figure 1; Supplementary Table 3). In almost every region, the mean cost per patient and ICU admission was significantly higher in COVID-19 patients.

## 4 Discussion

In our national study we found that 2.06% of pregnant women at the time of childbirth had COVID-19 infection. These women presented higher LoS, 4 times higher ICU admission rates, and 45 times higher ventilation/intubation rates. Although in-hospital mortality was quite infrequent (0.06%), it was 1,500 times higher compared to non-COVID-19 pregnant women. Regarding complications, we found higher rates of spontaneous premature onset of labor and postpartum hemorrhage, as well as higher induction of labor rates. Outcomes also showed a higher rate of single stillbirth (0.73%). Critically ill COVID-19 patients could lead to fetal hypoxia and stillbirth (15), which explains the need for labor induction in these cases. These results align with previous studies that found higher morbidity and mortality among pregnant women with COVID-19, as well as worse outcomes (16–21).

Regarding comorbidities, obesity or at least one risk factor have been associated with an increased risk of maternal death and ICU admission (22). In our study, both groups exhibited similar rates of risk factors in general, without any risk factor to be associated with an increased risk of COVID-19 in the multivariate analysis.

Numerous studies have examined both general and pregnancy-related complications associated with COVID-19. Several have reported an increased risk of gestational diabetes (23–25) while several reviews and meta-analyses have indicated a higher risk of preeclampsia (26, 27) in pregnant women infected with SARS-CoV-2. In contrast, our findings revealed an elevated risk of acute respiratory distress syndrome and embolism, which is consistent with the increased incidence of thrombotic events observed in these patients (28).

Furthermore, prior studies have reported higher rates of premature rupture of membranes (29, 30) preterm delivery (31, 32) and stillbirth (30–32) which are consistent with our observations. A national study conducted in England reported a lower prevalence of COVID-19 at childbirth (1.03%) and stillbirth (0.34%), but a higher rate of preterm deliveries (12.1%) compared to our data. That study also identified a stronger association of stillbirth (OR = 2.21) and preterm deliveries (OR = 2.17) with COVID-19 (33). Although we did not find a stronger association with preterm delivery, our results showed an increased risk of postpartum hemorrhage and stillbirth in women with COVID-19. These findings are consistent with prior reports and may be linked to inflammatory responses, coagulopathy, and changes in the placenta (34).

These discrepancies may be attributable to differences in study periods, particularly as our analysis involved three years since the beginning of the pandemic, during which different variants have circulated. Although vaccination status was not available for the participants in our study, COVID-19 vaccination during pregnancy has been shown to be safe and may help reduce risks and adverse outcomes (35).

Previous studies have assessed the direct costs of COVID-19 care in different contexts (9, 36, 37). However, this is the first study focusing on the increase in costs that COVID-19 can result in during hospitalization at childbirth. We found a higher mean cost per patient in general admission (3,820.32 €) as well as for ICU admission (13,629.35 €) in COVID-19 women compared to those admitted without COVID-19. The increased cost of COVID-19 at childbirth is likely related to the higher risk of complications and poorer outcomes in this specific population. Therefore, costs would involve longer stays and different treatment approaches required for those with COVID-19 which would entail higher resource use. These differences have remained consistent over the years. A higher mean cost was found in 2021 (5,149.72 €) for COVID-19 pregnant patients, which could be explained by the emergence of the Delta variant, which led to an increase in severity of cases (38, 39).

Additionally, the mean cost by age showed an increasing trend peaking in the population over 44 years old, with a mean cost per patient of 6,492.12€. The ICU admission rate also peaks in the over 44 years old group, reaching an 8.09%. These findings align with previous studies suggesting that older age is a risk factor for illness severity in pregnant women with COVID-19 (5, 6, 18). Finally, all regions showed a significant increase in the mean cost per patient for COVID-19 women at childbirth compared to those without COVID-19.

Our study is not without limitations. First, this is a retrospective study based on the Spanish MBDS; therefore, there could be coding errors and missing data. However, COVID-19 codes have been shown to have high sensitivity and specificity in other countries (40). Also, due to its retrospective nature, confounding factors were uncontrolled, and causal inference cannot be established. Second, cost estimates in the MBDS are calculated through diagnosis-related groups, a well-stablished American cost-estimation system (41). Third, the data is available with a two-year lag, therefore only data up to 2022 is available and this could change with new COVID-19 variants.

On the other hand, the strengths of our study include its nationwide scope, covering all childbirths recorded in the Spanish population over three years. The extensive data collected by the MBDS allowed us to evaluate the impact of COVID-19 on pregnant women at childbirth from multiple perspectives including the economic impact which to our knowledge has not been previously addressed.

To conclude COVID-19, affecting 2.06% of women at childbirth was associated with increased morbidity and mortality, including higher ICU admission rates, LoS and ventilation/intubation rates. It was also associated with complications related to pregnancy and childbirth and outcomes, such as spontaneous premature onset of labor, postpartum hemorrhage, higher induction of labor rates, and single stillbirth. The cost of these outcomes represents a 25.06% increase in the mean expenditure per patient. These data are related to SARS-CoV-2 variants circulating from 2020–2022, and current variants could give different risks. However, these differences underscore the need for specific preventive measures, such as vaccination and screening, and for strengthening resources to care for this vulnerable population. The findings provide a solid foundation for future public health strategies and clinical management during pandemics.

## Data Availability

Publicly available datasets were analyzed in this study. This data can be found at: the datasets presented in this article are not readily available because the MDBS is the property of the Ministry of Health. Therefore, any researcher can request the data related to this article from the Ministry of Health by email (icmbd@msssi.es), by fax (+34915964111), or by mail (Instituto de Información Sanitaria, Área de Información y Estadísticas Asistenciales, Ministerio de Sanidad, Consumo y Bienestar Social. Paseo del Prado 18–20; 28071 Madrid, Spain). Requests to access the datasets should be directed to Ministry of Health by fulfilling an application detailing the ICD-10 codes and periods needed.
