# Supplementary material for: Nationwide analysis of COVID-19 complications, outcomes, and costs of childbirth in Spain
Source: Front Med (Lausanne). 2025 May 8;12:1548245. doi: 10.3389/fmed.2025.1548245 (PMC12101083; doi:10.3389/fmed.2025.1548245)

Supplementary Material

1 Supplementary Figures and Tables

1.1 Supplementary Tables

Supplementary Table 1. ICD-10-CM codes used for comorbidities and complications.

Comorbidities	ICD-10-CM code
Asthma	J44.0. J44.1. J45
Diabetes	E08-E11
Chronic hypertension	I10
Chronic heart disease	H35.031-039. I01. I09.2. I05. I06. I34. I35. I36. I37. I09. I11. I12. I13. I21. I24. I25.2. I20. I25.700-I25.799. I26. I27. I28. I33. I30. I31. I38. I39. I42. I46. I47. I48. I49. I50. I40. I41. I51. I52. I70. I71. I72. I73. I74. I75. M30.0. M31. I77. Q21. Q22. Q23. Q24. Q25. Q26. Q27. T82. Z95
Metabolic disorder	E03-E07. E22. E23. E32. E27. E40-E46. E50-E56. E70-E72. E74. E78. E88. M10. E83
Immunosuppression	B20. B33.3. B59. B80. B81. B82. B73.1. D73. G70.8. M32.10. M35. M05. M06. R82.8. R84.7. R85.7. R86.7. R87.7. R89.7. T45. Z21. Z94
Blood disorder	D58. D59. D61. D64.0-3. D63.1. D63.0. D63.8. D64.81. D68. D69. D70. D71. D72. D73. D74. D75. D77
Neurologic disease	A17. A81. B90.0. F01. F02. F03. F07. F71. F72. F73. E75. G20. G25. G26. G11. M47. G60. G35. G36. G81. G80. G82. G40. G93. G96. I02. I60. I61. I62. I65. I66. I67.82. I67.9. I69. G71.13. R56. T85
Renal disease	D63. I12.0. I13.11. I13.2. N04. N03. N05. N17. N18. N19. N26.9. N25.0. N25.1. N27.0. N27.1. N27.9. N28.89. Q61. T85. Z49. Z91.15
**Pregnancy complications**	
Preeclampsia and eclampsia	O10.011-O10.013. O10.019. O10.02. O10.911-O10.913. O10.919. O10.92. 013. 014. O15.00, O15.02, O15.03, O15.1, O15.2, O15.9 O14.2
Gestational diabetes	O24
Premature rupture of membranes	O42
Antepartum hemorrhage	O46
Abortion	O03
Spontaneous premature onset of labor	O60.1x O60.2x
Induction of labor	O60.12X0. O60.13X0. O60.14X0
Caesarean section	O82. O82.0. O82.1. O82.2. O82.8. O82.9
Postpartum hemorrhage	O72
**Other complications**	
Acute renal failure	N17.0, N17.1, N17.2, N17.8, N17.9, O90.4
Acute respiratory distress syndrome	J80, J95.1, J95.2, J95.821, J95.822, J96.00, J96.01, J96.02, J96.20, J96.21, J96.22, R09.2
Embolism	I26.01, I26.02, I26.09, I26.90, I26.92, I26.99 O88.011-O88.019, 088.02, O88.03, O88.211-O88.219, O88.22, O88.23, O88.311-O88.319, O88.32, O88.33, O88.81, O88.82, O88.83
Temporal tracheostomy	0B110Z4, 0B110F4, 0B113Z4, 0B113F4, 0B114Z4, 0B114F4
Shock	O75.1, R57.0, R57.1, R57.8, R57.9, R65.21, T78.2XXA, T88.2XXA , T88.6XXA, T81.10XA, T81.11XA, T81.19X
Ventilation	5A1935Z, 5A1945Z, 5A1955Z
**Outcomes**	
Single livebirth	Z37.0
Single stillbirth	Z37.1
Twins. both liveborn	Z37.2
Twins. one liveborn and one stillborn	Z37.3
Twins. both stillborn	Z37.4

Supplementary Table 2. Univariant and multivariant regression results

	Univariant p-value	Multivariant p-value
**Comorbidities**		
Asthma	0.5	
Diabetes	0.566	
Chronic hypertension	0.603	
Chronic heart disease	<0.0001	0.91115
Metabolic disorder	0.148	
Immunosuppression	0.631	
Blood disorder	0.0093	0.10235
Neurologic disease	0.00077	0.89236
Renal disease	0.00918	0.21689
**Pregancy Complications**		
Preeclampsia/Eclampsia	0.365	
Gestational diabetes	0.328	
Premature rupture of membranes	0.00575	0.01649
Antepartum hemorrhage	0.835	
Abortion	0.43	
Spontaneous premature onset of labor	<0.0001	0.55732
Induction of labor	<0.0001	0.10607
Caesarean section	0.0287	0.03052
Postpartum hemorrhage	0.00027	0.00329
**Other complications**		
Acute renal failure	<0.0001	0.45564
Acute respiratory distress syndrome	<0.0001	<0.0001
Embolism	<0.0001	<0.0001
Temporal tracheostomy	<0.0001	0.00402
Ventilation/intubation	<0.0001	<0.0001
Shock	<0.0001	0.00029
**Outcomes**		
Single livebirth	0.899	
Single stillbirth	0.0002	0.02282
Twins, both liveborn	0.0538	
Twins, one liveborn and one stillborn	0.122	
Twins, both stillborn	0.628	

Supplementary Table 3. Mean cost (€) per patient and ICU admission rate of hospitalization at delivery based on COVID-19 infection in Spanish regions during 2020-2022.

Region	COVID-19 at delivery			Non-COVID-19 at delivery				
	n	Mean cost/patient (CI95%)	ICU admission n(%)	n	Mean cost/patient (CI95%)	ICU admission n(%)	P-valor cost	P-valor ICU
Andalucía	2413	4190.61 (4033.4-4347.84)	68 (2.82)	144344	3274.46 (3271.5-3277.44)	264 (0.18)	<0.0001	<0.0001
Aragón	378	4040.945 (3861.62-4220.26)	9 (2.38)	21887	3214.64 (3204.82-3224.48)	52 (0.24)	<0.0001	<0.0001
Principado de Asturias	156	3687.82 (3502.41-3873.23)	12 (7.69)	11208	3209.35 (3199.96-3218.74)	66 (0.59)	<0.0001	<0.0001
Illes Balears	261	3992.37 (3777.76-4206.98)	3 (1.15)	19665	3170.00 (3163.05-3176.95)	53 (0.27)	<0.0001	0.045
Canarias	610	3787.36 (3684.66-3890.07)	6 (0.98)	29720	3297.01 (3290.32-3303.7)	41 (0.14)	<0.0001	0.0004
Cantabria	151	3756.33 (3562.83-3949.84)	3 (1.99)	9004	3232.25 (3219.94-3244.57)	99 (1.1)	<0.0001	0.3525
Castilla y León	801	3797.65 (3702.53-3892.78)	16 (2)	35449	3250.12 (3244.66-3255.59)	244 (0.69)	<0.0001	0.0003
Castilla - La Mancha	876	4247.70 (3963.72-4531.69)	24 (2.74)	32086	3253.10 (3246.97-3259.24)	218 (0.68)	<0.0001	<0.0001
Cataluña	3493	4233.41 (4094.01-4372.82)	112 (3.21)	118724	3287.60 (3284.34-3290.87)	328 (0.28)	<0.0001	<0.0001
Comunitat Valenciana	1330	4004.38 (3907.01-4101.77)	27 (2.03)	79031	3277.02 (3272.54-3281.51)	446 (0.56)	<0.0001	<0.0001
Extremadura	261	3776.37 (3630.64-3922.11)	1 (0.38)	19395	3242.78 (3236.07-3249.5)	86 (0.44)	<0.0001	0.8815
Galicia	384	3894.21 (3738.07-4050.36)	16 (4.17)	38234	3236.55 (3230.83-3242.28)	879 (2.3)	<0.0001	0.0291
Comunidad de Madrid	2660	4097.84 (3954.55-4241.13)	61 (2.29)	109360	3234.72 (3231.45-3237.99)	1651 (1.51)	<0.0001	0.0024
Región de Murcia	824	3885.15 (3633-4137.3)	14 (1.7)	34749	3211.31 (3206.55-3216.09)	20 (0.06)	<0.0001	<0.0001
Comunidad Foral de Navarra	287	3939.9 (3372.47-4507.33)	7 (2.44)	12992	3200.82 (3192.02-3209.63)	47 (0.36)	<0.0001	0.0002
País Vasco	658	3800.55 (3675.87-3925.23)	14 (2.13)	36780	3166.76 (3161.63-3171.89)	148 (0.4)	<0.0001	<0.0001
La Rioja	105	3746.87 (3637.17-3856.57)	0 (0)	6334	3232.98 (3220.26-3245.7)	15 (0.24)	<0.0001	------
Ceuta	44	4198.2 (3475.84-4920.56)	3 (6.82)	2049	3295.55 (3272.23-3318.88)	7 (0.34)	<0.0001	0.0007
Melilla	50	4237.84 (3512.12-4963.56)	2 (4)	2634	3213.48 (3191.03-3235.94)	6 (0.23)	<0.0001	0.0072

ICU= Intensive care unit

Supplementary Figure 1. Mean cost per patient (€) of hospitalization at delivery based on COVID-19 infection in different Spanish regions. The one on the top represents non-COVID-19 women and the one on the bottom COVID-19 women at childbirth.

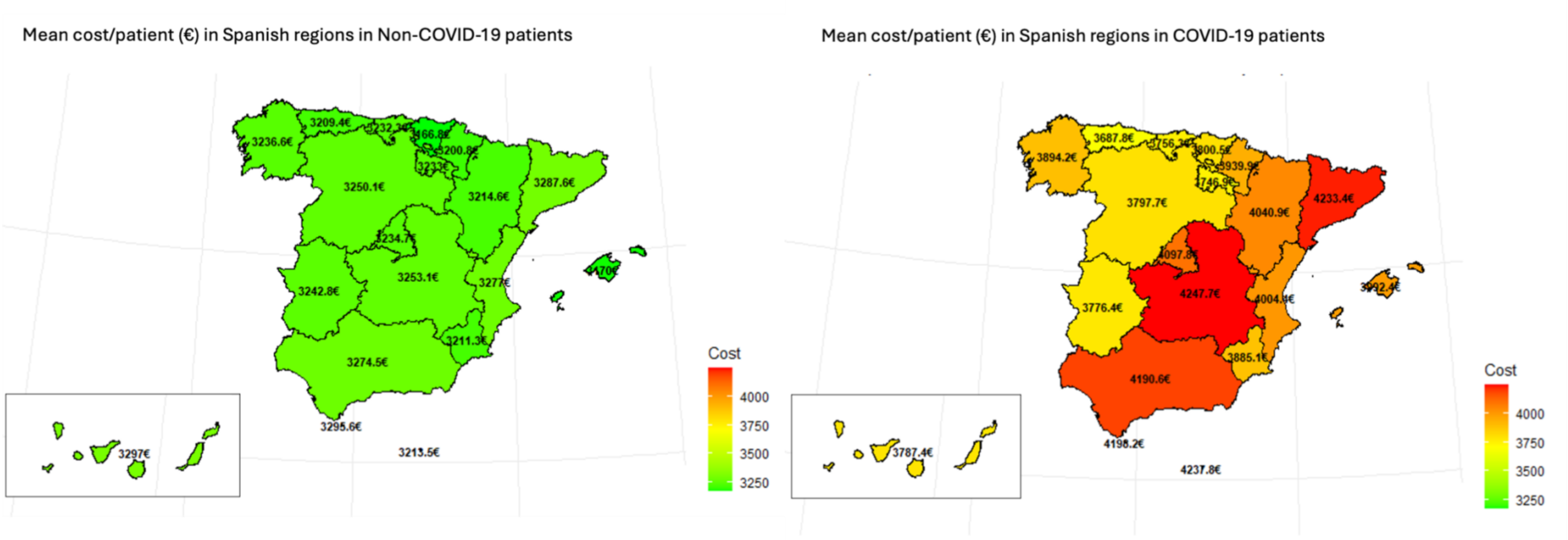

Supplement: Supplementary file 1 [file Data_Sheet_1.pdf]
